# Impacts of *Carpobrotus edulis* (L.) N.E.Br. on the Germination, Establishment and Survival of Native Plants: A Clue for Assessing Its Competitive Strength

**DOI:** 10.1371/journal.pone.0107557

**Published:** 2014-09-11

**Authors:** Ana Novoa, Luís González

**Affiliations:** Departamento de Bioloxía Vexetal e Ciencias do Solo, Facultade de Bioloxía, Universidade de Vigo, Vigo, Spain; Wuhan University, China

## Abstract

Does *Carpobrotus edulis* have an impact on native plants? How do *C. edulis’* soil residual effects affect the maintenance of native populations? What is the extent of interspecific competition in its invasion process? In order to answer those questions, we established pure and mixed cultures of native species and *C. edulis* on soil collected from invaded and native areas of Mediterranean coastal dunes in the Iberian Peninsula. We examined the impact of the invader on the germination, growth and survival of seeds and adult plants of two native plant species (*Malcolmia littorea* (L.) R.Br, and *Scabiosa atropurpurea* L.) growing with ramets or seeds of *C. edulis*. Residual effects of *C. edulis* on soils affected the germination process and early growth of native plants in different ways, depending on plant species and density. Interspecific competition significantly reduced the germination and early growth of native plants but this result was soil, density, timing and plant species dependent. Also, at any density of adult individuals of *C. edulis*, established native adult plants were not competitive. Moreover, ramets of *C. edulis* had a lethal effect on native plants, which died in a short period of time. Even the presence of *C. edulis* seedlings prevents the recruitment of native species. In conclusion, *C. edulis* have strong negative impacts on the germination, growth and survival of the native species *M. littorea* and *S. atropurpurea*. These impacts were highly depended on the development stages of native and invasive plants. Our findings are crucial for new strategies of biodiversity conservation in coastal habitats.

## Introduction

Invasive alien plants are considered as one of the greatest threats to the diversity, structure and functioning of natural ecosystems around the world [1], [2]. They can exert significant impacts on many ecological variables [3]. In particular, Mediterranean coastal dune ecosystems are highly sensitive to invasion by exotic plants since the disruption caused by the movement of sand constantly produces open spaces that are susceptible to colonization by alien species [4]. In those open spaces, competitive interactions between invasive and native species are extremely important [5]. As Mediterranean coastal dune ecosystems present a high cultural and ecological value, and support many threatened and endemic species [6], efforts on their alien species management strategies are critically needed.

One of the major invaders of Mediterranean coastal dune ecosystems is *Carpobrotus edulis* (L.) N.E.Br., a perennial clone plant native to South Africa [7]–[9]. Since its introduction, this invasive succulent now dominates millions of kilometres of Mediterranean dune ecosystems, leading to loss of species and irreversible changes on the substrate [7]–[10]. However, there is little information about the competitive interactions between this invasive species with native plants, even though it is a crucial aspect for prioritizing Mediterranean coastal dune ecosystems management.

Impacts of invasive species through competition with native plants is a primary ecological process limiting restoration success [11]. The phenologic stage of each species is decisive in these competitive relationships [12]: plants pass through different physiological stages and their development processes and competition occurs not only within species, but also within and between stages of different species [13]. However, most competition studies are focused on plants at the same development stage.

This study deals with the relative competitive ability of native seeds, seedlings and adult plants. It was designed to address the following questions: (i) the presence of *C. edulis* causes changes to soil characteristics (see Novoa et al. (2013a) for further information and detailed data). How do these changes affect the maintenance of native populations during each development stage? And (ii) To what extent are the ecological impacts caused by *C. edulis* based on different development stages of native plants? Despite the fact that the plant invasion process is a result of multiple interacting factors [14], [15], to the best of our knowledge this study is the first reporting simultaneous examination of multiple mechanisms: competition, density, timing of sowing, plant developmental stage and residual soil effects on the limitation of native flora by an invasive plant.

Moreover, since *C. edulis* can reproduce both vegetatively and sexually [16], understanding the competitive relationships established between clones or seeds of *C. edulis* and native species during the germination, establishment and growth processes are crucial for the conservation of the high biodiversity of Mediterranean coastal habitats [17].

## Materials and Methods

### 1. Plant material

The two main dominant native species in the study area were selected: a typical semi-fixed dunes species (*Malcolmia littorea* (L.) R.Br.) and a common fixed-dune and rocky species (*Scabiosa atropurpurea* L.) both of them Chamaephytes. Seeds of the native species *M. littorea* and *S. atropurpurea* and the invasive *C. edulis* were collected between 10 Sep and 10 Oct 2011 from at least 15 plants from 20 different populations of each species located along 20 km of the coast of Pontevedra, Spain (between 42°29′56.17″N 8°52′16.22″O and 42°20′16.22″N 8°49′41.17″O). The land accessed is not privately owned or protected. The seeds were separated from the rest of the fruit and its accessory dispersion parts and stored in the dark at 4°C until assay. Seeds were surface-sterilized for 5 min in 0.1% sodium hypochlorite, rinsed 3 times in distilled water and dried at room temperature.

**Figure 1:**
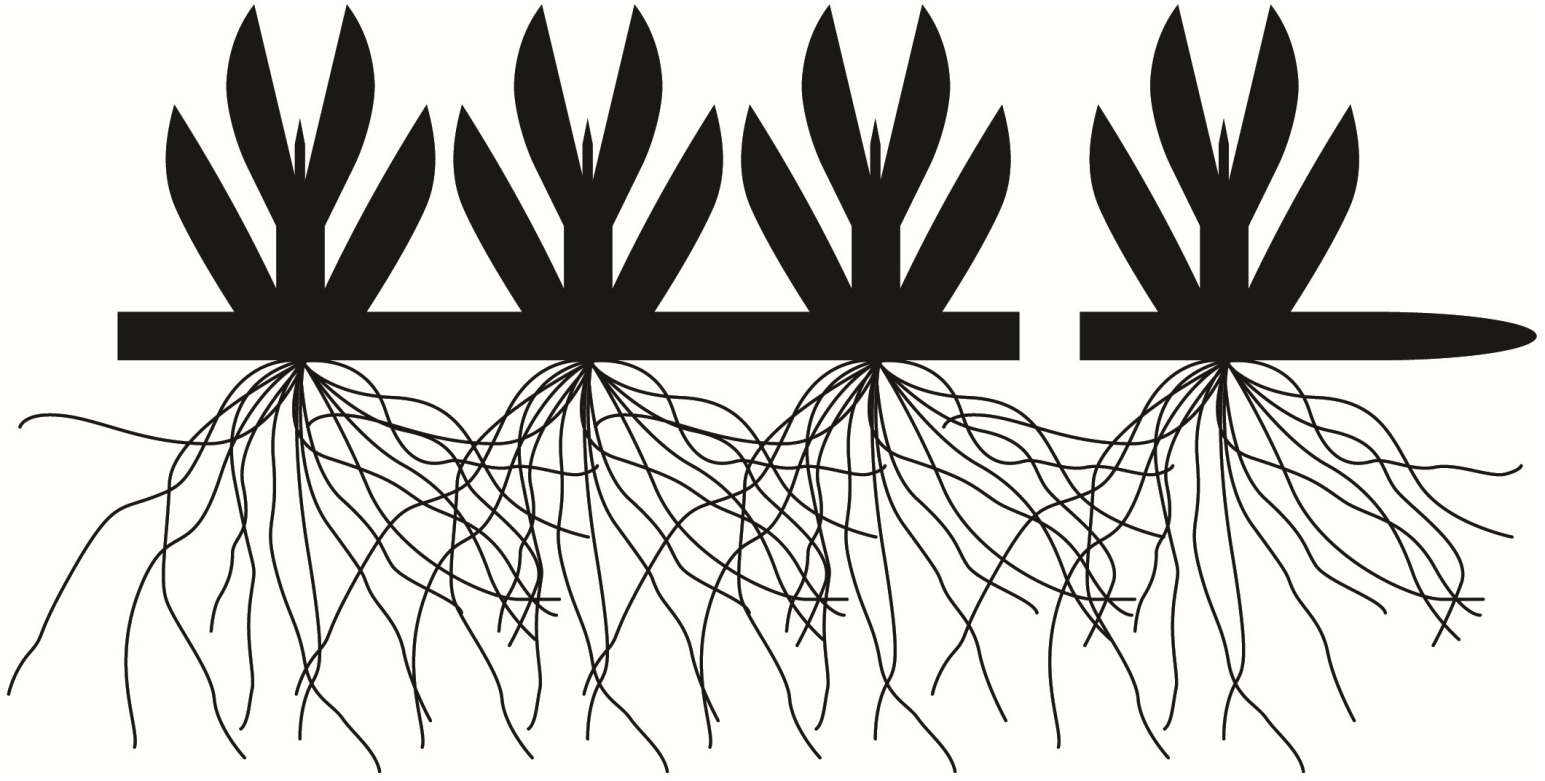
Collection of adult plants of *C. edulis.*

On the 19 Nov 2010, adult plants of native species (*M. littorea* and *S. atropurpurea*) and apical ramets of the invasive *C. edulis* -as well as a volume of 1 L of sand around each plant- were collected in the same area and immediately transplanted to 1 L sand pots for greenhouse acclimatization, integrating the plant stock. As *C. edulis* presents clonal growth, we could obtain individuals with approximately the same developmental state from the ends of the branches (Figure 1).

### 2. Soil collection

On the 19 Nov 2010, soil was collected up to 10 cm depth in those dunes from where the seeds, adult plants and ramets had previously been collected. The top soil layer from 20 randomly selected points (1×1 m) was collected in an area invaded by *C. edulis* and in an adjacent native area. The adjacent native area was sufficiently separated from the invaded area, to affirm that there was no effect of *C. edulis* on the soil. The soil taken from each area (invaded or native) was homogenized (approx. 100 Kg) and reserved for the establishment of the crops, as explained later. See [8] for physicochemical results.

### 3. Competition between *C. edulis* ramets and native species

In order to look for competitive relationships established between *C. edulis* and native plants in different stages of native plant’s development (germination, seedling and adult plant), pot culture experiments were established in both invaded and native soil following the principle of replacement or substitution [18], modified for our purposes (Table 1). Competition experiments were carried out on two soils with different origins (native and invaded soil). To avoid interference in the replacement series due to physico-chemical and biological characteristics of the soil, a concomitant treatment checking intra-specific competition density was established. Four experimental trials were then established to study the competitive interaction between the invasive *C. edulis* and native species: (a) intra-specific competition between native seeds/seedlings (b) ramets of *C. edulis vs* native seeds/seedlings, (c) intra-specific competition between native adult plants and (d) ramets of *C. edulis vs* adult native plants. Cultures were established in 1 L pots filled with soil from native and invaded zones and replicated five times. A total of 320 pots were used.

**Table 1:** Methodological scheme for greenhouse experiment.

		Ramets of *C. edulis*				
		*0*	*1*	*2*	*3*	*4*
N° of native species seeds or seedlings	***0***	•				X
	***10/1***	•			X	
	***15/2***	•		X		
	***20/3***	•	X			
	***25/4***	X				

(X) Represent classic replacement series design. (•) Represent modified replacement series. N = 5.

#### 3.1. Intra-specific competition between native seeds and seedlings

80 pots with cultures containing 10 15, 20 or 25 seeds of the native species (*M. littorea* or *S. atropurpurea*) were established (Table 1), which we refer to in this paper as “pure seed cultures.” Total germination rate (Gt), cumulative rate of germination (AS) [19], survival and early growth were determined. The number of germinated seeds and plant survival were recorded daily for ten weeks.

#### 3.2. Ramets of *C. edulis vs* native seeds and seedlings

In order to check inter-specific competition, mixed cultures were established in 80 pots, combining 3, 2, 1 and 0 ramets of the invasive *C. edulis* with 10, 15, 20 and 25 seeds of native species, referred to as “mixed seed/ramet cultures” (10/3, 15/2, 20/1 and 25/0). Total germination rate (Gt), cumulative rate of germination (AS) [19], survival and early growth were determined. The number of germinated seeds and plant survival were recorded daily for ten weeks.

#### 3.3. Intra-specific competition between native adult plants

In order to take into account intra-specific competition, cultures containing 4, 3, 2 or 1 adult native plants (*M. littorea* or *S. atropurpurea*) were established (Table 1) in 80 pots, referred to as “pure adult cultures”. Leaf number, height and survival were recorded every three days for two weeks.

#### 3.4. Ramets of *C. edulis vs* adult native plants

In order to check inter-specific competition, mixed cultures with 3, 2, 1 and 0 ramets of the invasive *C. edulis* combined with 1, 2, 3 or 4 adult native plants were cultivated in 80 pots, referred to as “adult/ramets cultures” (1/3, 2/2, 3/1 and 4/0). Leaf number, height and survival were recorded every three days for two weeks.

### 4. Competition between *C. edulis* seeds and native seeds

Seeds of *C. edulis* and *M. littorea* or *S. atropurpurea* were sowed at different densities and times, following the scheme proposed by Tielbörger and Prasse [5]. Five replicates of the following seed mixture were established: 10 seeds of each native species plus 10 seeds of *C. edulis*, 10 seeds of each native species plus 30 seeds of *C. edulis*, 30 seeds of each native species plus 10 seeds of *C. edulis* and pure crops of 30 or 10 seeds of each species. With the aim of testing the effect of time, the seeds of *C. edulis* were sowed at different date ranges (5 days before the native species, at the same time, or 5 days later than the native species). Competition conditions were established in Petri dishes filled with soil from invaded and from native areas. A total of 240 petri dishes were used. The experimental design (Table 2) therefore had 5 independent factors: neighbour density (10 vs 30), target species (two native species), neighbour species (one invasive species), soil type (native or invaded) and timing of sowing (5 days before, at the same time or 5 days latter).

**Table 2:** Methodological scheme for growth chamber experiment.

	Day 0		Day 5				Day 10
	*5 days before sowing native species*		*Sowing date of native seeds*				*5 days after sowing native species*
	Seeds number						
	*C. edulis*	native		*C. edulis*	native		*C. edulis*
Assay 1	10		+10				
	10		+30				
	30		+10				
Assay 2			10+	10			
			30+	10			
			10+	30			
Assay 3					10	+	10
					30	+	10
					10	+	30
Monocultures		10		10			
		30		30			

Assay 1: Interspecific competition, *C. edulis* seeds sowed before native seeds. Assay 2: Interspecific competition, *C. edulis* seeds sowed at the same time that native seeds. Assay 3: Interspecific competition, *C. edulis* seeds sowed after native seeds. Monocultures: intraspecific competition for native and *C. edulis* seeds. N = 5.

The Petri dishes were incubated in germination chambers with periods of 12 hours of light and 25°C/15°C, temperatures and light regimes similar to those in the field. Substrate moisture in sandy soils is one of the most limiting factors of plant growth [20]. Therefore, all of the seeds were watered every two days, as previous trials have indicated that this procedure permits maximum germination despite the limited amount of substrate. Percolation of the water through holes in the bottom of the dishes was allowed, avoiding the formation of a salt crust. The number of germinated seeds and plant survival were recorded daily for ten weeks. Total germination rate (Gt), and the cumulative rate of germination (As) were determined [19]. After approximately ten weeks of watering, no further germination was observed and the length of leaf, stem and roots of 7 seedlings per plate and species were measured.

### 5. Statistical analysis

The Kolmogorov–Smirnov test and Levene’s test were used to ensure the normality assumption and the homogeneity of variances, respectively. Two-way analysis of variance (ANOVA) was performed to assess the significance of the effects of soil characteristics and density as well as of their interaction on the studied parameters of germination, growth and survival. Tukey’s test was applied for all post-hoc comparisons between groups.

A two-way ANOVA involving soil characteristics and density as factors was carried out to detect significant differences between treatments for each native species. All statistical analyses were performed using the IBM SPSS Statistic 19.0 (IBM, Armonk, NY, USA) software package.

## Results

### 1. Competition of *C. edulis* ramets *vs* native species

#### 1.1. Intra-specific competition between native seeds and seedlings

In invaded soil, pure seed cultures of *M. littorea* (10, 15, 20 and 25 seeds) showed a significant decrease in the total germination percentage (up to 56.4%) and cumulative rate of germination (up to 55.1%) with the increase in density (Table 3). However, in native soil there were no significant differences between treatments (seed number). It is quite remarkable that at the highest density (25 seeds), the total germination and cumulative rate of germination were significantly greater on native soil compared with invaded soil (*P*≤0.05), while at low seed densities (10, 15 and 20 seeds) we observed the opposite trend (Table 3). The survival percentage of seedlings of *M. littorea* showed a significant increase (21.74%) at high densities in native soil. The shoot and root length of *M.* seedlings in both soils showed no significant effect of intra-specific competition between treatments (number of individuals). Despite this, the growth values from invaded soil are significantly higher than the lengths in the native soil (*P*≤0.05).

**Table 3:** Intraspecific competition effect on the germination indices (Gt: Total germination and AS: Cumulative rate of germination), seedling shoot and root length and survival of the native species *Malcolmia littorea* in both invaded and native soil.

	Invaded soil				Native soil			
Seed number	10	15	20	25	10	15	20	25
Gt	82.5^a^*	81.7^a^*	57.0^b^	36.0^c^*	51.2	62.3	53.0	65.6
	(9.8)	(9.6)	(6.8)	(5.7)	(4.0)	(3.4)	(6.4)	(3.4)
AS	105.8^ab^*	112.9^a^	84.3^b^*	47.8^c^*	67.2	82.7	54.8	88.5
	(14.1)	(10.5)	(6.3)	(3.8)	(8.6)	(10.7)	(12.6)	(43)
Survival (%)	71.0	94.1	82	77.9	72^b^	81^b^	79^b^	92^a^
	(12.7)	(11.9)	(14.3)	(17.6)	(2.4)	(9.6)	(6.3)	(2.8)
Shoot length (cm)	1.2*	1.5*	1.4*	1.3*	0.9	1.1	0.9	1.1
	(0.1)	(0.2)	(0.1)	(0.1)	(0.1)	(0.1)	(0.1)	(0.1)
Root length (cm)	2.3*	2.3*	2.3*	2.3*	1.4	1.6	1.4	1.5
	(0.2)	(0.3)	(0.2)	(0.2)	(0.1)	(0.1)	(0.1)	(0.1)

Different letters mean significant difference of 5% between treatments (10, 15, 20 or 25 seeds).*: indicates significant difference of 5% between invaded and native soils on each treatment. Numbers in parentheses indicate the standard error. N = 5.

No significant effect of intra-specific competition was found between *S. atropurpurea* seedlings affecting the growth, survival or germination rates (Table 4).

**Table 4:** Intraspecific competition effect on the germination indices (Gt: Total germination and AS: Cumulative rate of germination), seedling shoot and root length and survival of the native species *Scabiosa atropurpurea* in both invaded and native soil.

	Invaded soil				Native soil			
Seed number	10	15	20	25	10	15	20	25
Gt	64.0	53.3	43.0	45.6	44.0	49.3	40.0	48.0
	(11.7)	(9.2)	(8.6)	(2.7)	(10.3)	(6.5)	(8.2)	(7.2)
AS	51.0	41.7	33.6	40.1	42.7	39.9	35.8	46.1
	(5.7)	(4.0)	(5.0)	(2.9)	(5.1)	(3.4)	(6.5)	(1.9)
Survival (%)	80.2	87.7	90.2	93.5	68.8	84.7	89.0	94.5
	(11.1)	(4.8)	(4.9)	(1.7)	(12.8)	(4.6)	(3.5)	(2.5)
Shoot length (cm)	0.9^b^	1.2^ab^	1.1^ab^	1.4^a^	0.79	1.1	1.0	1.2
	(0.1)	(0.2)	(0.2)	(0.2)	(0.1)	(0.2)	(0.1)	(0.2)
Root length (cm)	5.3^b^	5.6^b^	5.3^b^	6.7^a^	4.5^b^	5.2^b^	5.4^b^	7.1^a^
	(0.7)	(0.4)	(0.2)	(0.6)	(0.9)	(0.4)	(0.3)	(0.7)

Different letters mean significant difference of 1% between treatments (10, 15, 20 or 25 seeds). Numbers in parentheses indicate the standard error. N = 5.

#### 1.2. Ramets of *C. edulis vs* native seeds and seedlings

The increase in density of *C. edulis* per pot did not affect the germination process of *M. littorea* in invaded soil. However, in native soil, the higher density of *C.* caused a decrease in the percentage and speed of germination of *M. littorea* (78% and 87% respectively). The survival of *M. littorea* seedlings was lower as the density of *C. edulis* increased, and was null in native soil (Table 5). Most of the treatments with a high density of *C. edulis* showed higher germination rates and survival percentages in invaded than in native soil. The opposite situation was shown for germination rates when *C. edulis* was not present. It was impossible to test the early seedling response of *M. littorea* experimentally, due to the low or null survival percentage.

**Table 5:** Inter-specific competition effect on the germination indices (Gt: Total germination and AS: Cumulative rate of germination), seedling shoot and root length and survival of the native species *Malcolmia littorea* on both invaded and native soil.

	Invaded soil				Native soil			
Seed N°/ramets	10/3	15/2	20/1	25/0	10/3	15/2	20/1	25/0
Gt	46.0*	33.3*	37.0	36.0*	15.8^c^	26.7^bc^	45^ab^	65.6^a^
	(13.3)	(9.7)	(12.9)	(5.7)	(5.5)	(3.8)	(13.5)	(3.4)
AS	51.5*	42.1	38.0	47.8*	12.8^c^	23.6^bc^	44.9^b^	88.5^a^
	(10.8)	(8.6)	(10.5)	(3.8)	(7.4)	(5.8)	(15.2)	(43)
Survival (%)	32.5^b^*	18.6^b^	51.8^ab^*	77.9^a^	0^b^	0^b^	92^a^	(2.8)
	(12.8)	(9.9)	(16.0)	(17.6)				
Shoot length (cm)	-	-	-	-	-	-	-	-
Root length (cm)	-	-	-	-	-	-	-	-

Different letters mean significant difference of 5% between treatments (10/3, 15/2, 20/1 or 25/0 seeds/ramets).*: indicates significant difference of 5% between invaded and native soils on each treatment. Numbers in parentheses indicate the standard error. N = 5.

The germination process and early growth of *S. atropurpurea* were not affected by the increase in density of *C. edulis* and slightly affected by the type of soil (Table 6).

**Table 6:** Inter-specific competition effect on the germination indices (Gt: Total germination and AS: Cumulative rate of germination), seedling shoot and root length and survival of the native species *Scabiosa atropurpurea* on both invaded and native soil.

	Invaded soil				Native soil			
Seed N°/ramets	10/3	15/2	20/1	25/0	10/3	15/2	20/1	25/0
Gt	30.0	41.3	37.1	45.6	14.0	24.0	37.0	48.0
	(8.9)	(3.9)	(3.7)	(2.7)	(9.8)	(7.5)	(3.0)	(7.2)
AS	23.2*	28.3	32.8	40.1*	3.8	19.8	30.4	46.1
	(7.0)	(2.7)	(4.7)	(2.9)	(7.9)	(6.1)	(1.7)	(1.9)
Survival (%)	31.1*	68.3	79.8	93.5	70.0	51.4	51.7	94.5
	(18.1)	(18.1)	(7.5)	(1.7)	(20.0)	(16.4)	(21.5)	(2.5)
Shoot length (cm)	1.2	1.5	1.1	1.4	1.7	1.2	1.2	1.3
	(0.2)	(0.5)	(0.2)	(0.2)	(0.3)	(0.2)	(0.2)	(0.2)
Root length (cm)	4.1	5.0	5.8	6.4	5.4	4.4	4.9	6.3
	(1.1)	(1.4)	(0.7)	(1.4)	(0.5)	(0.9)	(0.3)	(0.9)

Different letters mean significant difference of 5% between treatments (10/3, 15/2, 20/1 or 25/0 seeds/ramets).*: indicates significant difference of 5% between invaded and native soils on each treatment. Numbers in parentheses indicate the standard error. N = 5. None of the post-hoc ANOVA results were significant.

The interaction between the dependent factors (soil origin and density) was studied by a two-way ANOVA. The interaction was not significant, indicating that the internal relationship between soil origin and density in the germination process and early growth of native plants is very small, or does not exist.

#### 1.3. Intra-specific competition between native adult plants

In native pure adult cultures there were no significant differences in leaf number, height and survival between plant densities or any soil effect on the plant traits recorded (data not shown).

#### 1.4. Ramets of *C. edulis vs* adult native plants

**Figure 2:**
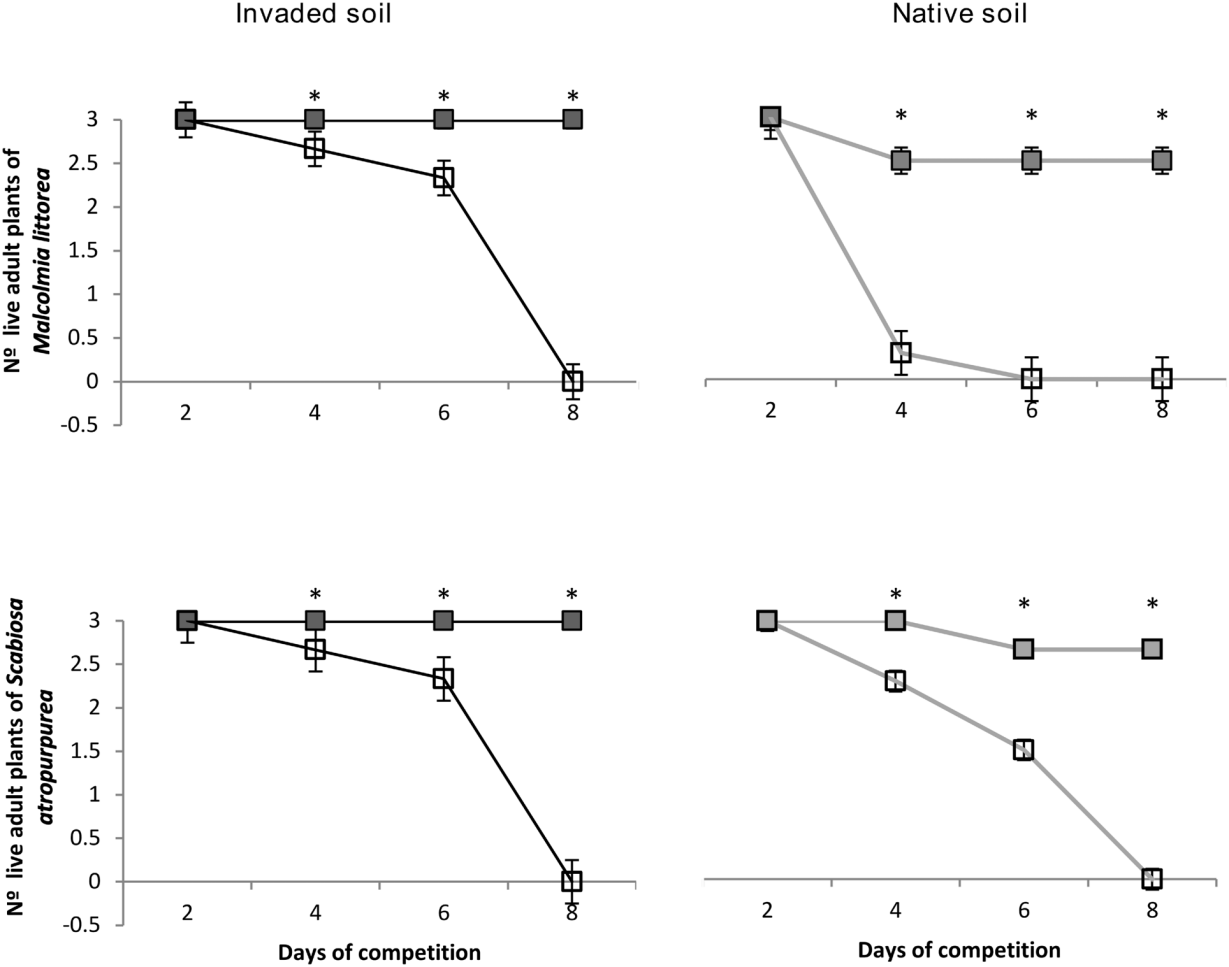
Survival of the native plants *Malcolmia littorea* and *Scabiosa atropurpurea* in pure adult cultures (3 adult native plants) and mixed adult/ramets cultures (3 adult native plants against 1 ramet of *C. edulis*) on invaded and native soil. *: indicate differences (*P*≤0.05) between monocultures (filled squares) and mixed (empty squares).

Mixed adult/ramet cultures revealed that the presence of the invasive plant *C. edulis* had a lethal effect on the adult plants of *M. littorea* and *S. atropurpurea*. All native plants died within 8 days of competition, even when the ratio for the invasive plant *C. edulis* was minimal (3 adult native plants against 1 ramet of *C. edulis*) (Figure 2).

### 2. *C. edulis* seeds *vs* native seeds

#### 2.1. Intra-specific competition and soil effects

Considering pure seed cultures, we found no density effect (*P*≤0.05) and almost not soil effect (Table 7). However, the Gt and AS indices of were in general higher in Petri dishes filled with invaded soil than in those filled with native soil while the shoot growth seemed higher on non-invaded soils (Table 7).

**Table 7:** Effect of soil on the germination and early growth of the invader *C. edulis* and the native species *Malcolmia littorea* and *Scabiosa atropurpurea* in monocultures.

		Gt		AS		Shoot growth		Radiclegrowth	
	N° seeds	I	N	I	N	I	N	I	N
*C. edulis*	10	30.0	23.3	0.19	0.16	0.49*	0.30	1.7*	2.6
		(10.0)	(8.8)	(0.09)	(0.09)	(0.08)	(0.07)	(0.1)	(0.3)
	30	38.9	22.2	0.21	0.12	0.53	0.38	2.1	2.6
		(4.0)	(5.9)	(0.04)	(0.01)	(0.07)	(0.04)	(0.2)	(0.38)
*Malcolmia* *littorea*	10	60.0	53.3	0.78	0.53	0.24	0.24	1.79	1.9
		(10.0)	(8.8)	(0.20)	(0.10)	(0.02)	(0.04)	(0.2)	(0.1)
	30	65.6	56.7	1.05*	0.54	0.25	0.22	2.1	2.5
		(1.9)	(10.0)	(0.02)	(0.09)	(0.02)	(0.01)	(0.1)	(0.4)
*Scabiosa* *atropurpurea*	10	46.7	73.3	0.73	0.99	0.35	0.33	8.1*	10.2
		(12.0)	(13.3)	(0.24)	(0.19)	(0.02)	(0.02)	(1.2)	(0.8)
	30	65.6	70.0	0.94	0.90	0.44*	0.29	12.0	10.4
		(5.9)	(5.1)	(0.09)	(0.10)	(0.04)	(0.01)	(0.6)	(0.7)

(Gt: Total germination and AS: Cumulative rate of germination). *indicate significant differences at 5% level between the seeds sown in Petri dishes filled with invaded soil and those sowed in native soil. Numbers in parentheses indicate the standard error. I: Invaded soil, N: Native soil. N = 5.

#### 2.2. Inter-specific competition and soil effects

In relation to time of sowing, the germination indices for *M. littorea* seeds were generally significantly lower when the seeds of *C. edulis* were sown 5 days before. Values were generally similar in assay 2 and 3 and in monocultures.

*C. edulis* seeds reacted the same way in the presence of *M. littorea* seeds in native soils. The germination indices were generally significantly lower when the seeds of *M. littorea* were sown 5 days before (80–90%) but not in the presence of *S. atropurpurea* (Table 8). The germination process of *S.* did not appear to be influenced by any of the treatments (Table 8).

**Table 8:** Effect of the timing of sowing on the germination of the invader *C. edulis* and the native species *Malcolmia littorea* and *Scabiosa atropurpurea littorea*.

		Gt				AS			
		A1	A2	A3	M	A1	A2	A3	M
CM	I	36.7^a^	26.7^b^	20.0^b^	30.0^a^	0.50	0.24	0.14	0.19
		(3.3)	(3.3)	(10.0)	(10.0)	(0.04)	(0.09)	(0.09)	(0.09)
	N	30.0^a^	3.3^b^	3.3^b^	23.3^a^	0.43^a^	0.02^b^	0.01^b^	0.16^a^
		(0.0)	(3.3)	(3.3)	(8.8)	(0.06)	(0.02)	(0.01)	(0.09)
CS	I	43.3	23.3	30.0	30.0	0.39	0.14	0.07	0.19
		(3.3)	(3.3)	(5.8)	(10.0)	(0.01)	(0.07)	(0.01)	(0.09)
	N	20.0	13.3	10.0	23.3	0.22	0.09	0.05	0.16
		(5.8)	(3.3)	(5.8)	(8.8)	(0.11)	(0.05)	(0.02)	(0.09)
ML	I	33.3^b^	70.0^a^	66.7^a^	60.0^a^	0.36^b^	1.01^a^	0.86^a^	0.78^a^
		(8.8)	(17.3)	(12.0)	(10.0)	(0.15)	(0.14)	(0.15)	(0.20)
	N	20.0^b^	30.0^a^	40.0^a^	53.3^a^	0.13^b^	0.30^a^	0.46^a^	0.53^a^
		(5.8)	(2.8)	(7.5)	(8.8)	(0.03)	(0.05)	(0.09)	(0.10)
SA	I	46.7	63.3	66.7	46.7	0.59	0.89	0.98	0.73
		(6.7)	(3.3)	(6.7)	(12.0)	(0.07)	(0.01)	(0.06)	(0.24)
	N	70.0	70.0	66.7	73.3	0.77	1.05	0.92	0.99
		(15.3)	(15.3)	(12.0)	(13.3)	(0.19)	(0.20)	(0.10)	(0.19)

Different letters means significant differences at 5% level among treatments (A1, A2, A3, M).

A1: Assay 1, *C. edulis* seeds sown before native seeds. A2: Assay 2: *C. edulis* seeds sowed at the same time that native seeds. A3: Assay 3: *C. edulis* seeds sown after native seeds. M: native monocultures. I: Invaded soil. N: Native soil. CM: *C. edulis* + *M. littorea*. CS: *C. edulis* + *S. atropurpurea.* ML: *M. littorea*. SA: *S. atropurpurea*. Numbers in parentheses indicate the standard error. N = 5.

The growth results in relation to the time of sowing factor only refer to native species, as *C. edulis* had different growth periods depending on the treatment (assay 1, 2 or 3). *M. littorea* seedlings showed a decrease in shoot growth with the presence of *C. edulis* seeds, especially in native soils, although the radicle growth of *M. littorea* showed no differences (Table 9). No effects were observed in *S. atropurpurea* growth in relation to the timing factor (Table 9).

**Table 9:** The effect of timing of sowing on the early growth of the native species *Malcolmia littorea* and *Scabiosa atropurpurea*.

		Shoot growth				Radicle growth			
		A1	A2	A3	M	A1	A2	A3	M
ML	I	0.20^b^	0.20^b^	0.17^b^	0.24^a^	2.1	1.79	1.7	1.79
		(0.00)	(0.00)	(0.02)	(0.02)	(0.5)	(0.2)	(0.2)	(0.2)
	N	0.24^b^	0.23^b^	0.17^b^	0.30^a^	1.9	1.9	1.7	1.9
		(0.00)	(0.03)	(0.02)	(0.04)	(0.1)	(0.3)	(0.3)	(0.1)
SA	I	0.34	0.35	0.40	0.35	10.9	10.0	10.5	8.1
		(0.03)	(0.02)	(0.03)	(0.02)	(1.3)	(0.6)	(1.0)	(1.2)
	N	0.35	0.32	0.28	0.33	9.0	11.1	10.7	10.2
		(0.03)	(0.02)	(0.03)	(0.02)	(1.1)	(1.1)	(1.0)	(0.8)

Different letters means significant differences at 5% level among treatments (A1, A2, A3, M).

A1: Assay 1, *C. edulis* seeds sown before native seeds. A2: Assay 2: *C. edulis* seeds sowed at the same time that native seeds. A3: Assay 3: *C. edulis* seeds sown after native seeds. M: native monocultures. I: Invaded soil. N: Native soil. ML: *M. littorea*. SA: *S. atropurpurea*. Numbers in parentheses indicate the standard error. N = 5.

Despite the effects of the time of sowing, there were no significant differences (*P*≤0.05) between pure and mixed cultures in any treatment (results not shown).

## Discussion

### 1. Competition between ramets of *C. edulis* and native species

#### 1.1. Intra-specific competition between native seeds and seedlings

Soil from habitats invaded by *C. edulis* had a markedly species-dependent effect. *M. littorea*, a typical semi-fixed dunes species, is a specialist plant of poor and slightly saline soils [21]. Its germination and growth process is significantly influenced by soil changes induced by *C. edulis* [7], [8]. In general, we found a reduction in the germination process as the density increased in invaded soil. The advantage of this behaviour of density-dependent germination in invaded soils is difficult to understand. It is possible that for these plants there is an advantage of not germinating under competitive conditions, so they may exploit more favourable conditions in later years [5]. Shoot and root length was always greater in invaded soils, probably due to a greater availability of nutrients in these soils [9], [22] and salt concentration [23] that stimulate growth. In native soil, the survival percentage increased at the highest density, which may be due to the nurse plant phenomenon [20] taking into account not differences between life forms.

Germination was not density dependent for *S. atropurpurea* in either invaded or native soils. As previously mentioned *S. atropurpurea* presented a greater plasticity to adapt to different soil conditions and seems to be less of a specialist than *M. littorea*.

#### 1.2. Ramets of *C. edulis vs* native seeds and native seedlings

Inter-specific competition is species-dependent and determines what species can coexist [24]. *C. edulis* differentially affected germination of the selected native plants. Soil characteristics became as one of the factors that directly affect the competitive potential of the exotic [25].

*M. littorea* showed a significant decrease in the germination process caused by the presence of *C. edulis* adult plants. However, the most dramatic effect found on *M. littorea* is in the survivorship, which is soil dependent. In invaded soil, the survival percentage decreases with the proportion of *C. edulis* adult plants in the mixed cultures, while in native soils, none of the germinated seedlings survived at the end of the bioassay. Inter-specific competition seems to be responsible for these results [26], [27]. Thus, although barriers to native plant germination could be overcome, *M. littorea* seedlings would not establish viable populations in the presence of *C. edulis* adult plants.

Soil characteristics are not determinant in the response of *S. artropurpurea* to the presence of *C. edulis* (except to mixed seed/ramets cultures 10/3), once again probably due to its plasticity. Although *C. edulis* threatens the establishment of *S. atropurpurea* in both soils in different ways, seedlings are phenotypically plastic in their allocation of biomass into roots and shoots [28].

#### 1.3. Intra-specific competition between natives adult plants

Our results indicate that the residual effect of *C. edulis* on dune soil does not affect the development of native adult plants. Adult native plants seem to be better adapted to soil changing conditions than seedlings, suffering stress from the residual effect in the soil.

#### 1.4. Ramets of *C. edulis vs* adult native plants

The presence of *C. edulis* in the replacement series had a deleterious effect on native plants independently of density and soil type. Different authors have indicated that allelopathy is a fairly common invasion mechanism (29). Ens et al. [30] proposed that the eventual dominance of invasive species could be explained by direct or indirect chemical inhibition of the establishment of indigenous plants, which was confirmed by Novoa et al. [7] for *C. edulis*.

### 2. Seed competition of *C. edulis vs* seeds of native species

#### 2.1. Soil effects

Dune species germinate in autumn, the rainy season, when in addition to having more water in the soil, the salt content decreases [20]. Water softens the seed coat so that the root can emerge more easily and also solubilizes nutrients [31]. A high salt content can block the germination process by the osmotic effect, drawing water from seeds [32]. During the assay, all of the seeds were watered every two days. However, invaded soils have a higher level of organic matter than native soils [7], [8], so they can hold water for a longer period of time. As a result, the Gt and AS indices of *M.* were higher in Petri dishes filled with invaded soil than in those filled with native soil. Also, in the presence of *C. edulis*, *M. littorea* has greater shoot growth in native soils. This could be due to an allelopathic effect of *C. edulis* seeds on *M. littorea* [7].

Once again *S. atropurpurea* did not show any differences in the germination process between soils. However, the radicle growth of *S. atropurpurea* seedlings seemed to be stimulated by native soil, while shoot growth seemed to be stimulated by invaded soil. The nutrient content of sand (especially nitrogen and phosphorus) positively or negatively affects the growth of dune species depending on the species [20]. The radical growth of *S. atropurpurea* decreases and its shoot growth increases with an increase in the nutrient level [8]. This could explain the differences observed in the growth of *S. atropurpurea* between invaded and native soil.

Finally, when *C. edulis* invades coastal habitats, it modifies the conditions of the substrate and suffers from difficulties as a result of tissue decomposition [33]. This feature could have evolved as a mechanism to facilitate recolonization when the clones die, and it influences the germination process of the invasive species. As a result, the germination process of *C. edulis* depends on the nutrient level of the soil [7]. Therefore, the Gt and AS indices of *C. edulis* were higher in Petri dishes filled with invaded soil than in those filled with native soil. Also, the radicle of *C. edulis* grows more at high pH levels [7], and so, it grew more in native than in invaded soils.

#### 2.2. Timing of sowing

We observed that the timing of sowing affects the establishment of both *C. edulis* and *M. littorea*, although there are no competitive interactions if they are sown at the same time. Therefore, they could have some mechanisms that allow their seeds to evaluate the conditions of their neighbours prior to emergence and to plastically respond to them [5]. *C. edulis* could also have an allelopathic effect on *M. littorea* and vice versa, which should be explored in future assays. These are important results to be taken into account for restoration actions; but also in conservation actions. If *C. edulis* is a new invader arriving from seeds into a native area and *M. littorea* is present, according to our results it appears that M. littorea might have the ability to supress *C. edulis* germination.

## Conclusion

Our results show that the impacts of *C. edulis* on native plants are highly dependent on its development stages as well as on the development stages of native plants. These findings are crucial for new strategies of biodiversity conservation in coastal habitats.
